# A Conscious Sedation Protocol for Videolaryngostroboscopy in Pediatric Patients

**DOI:** 10.1155/2010/643123

**Published:** 2010-11-29

**Authors:** Samantha Anne, Lawrence M. Borland, Laura Haibeck, Joseph E. Dohar

**Affiliations:** ^1^Department of Pediatric Otolaryngology, Children's Hospital of Pittsburgh, Pittsburgh, PA 15213, USA; ^2^Department of Otolaryngology, University of Pittsburgh School of Medicine, Pittsburgh, PA 15260, USA; ^3^Department of Pediatric Anesthesiology, Children's Hospital of Pittsburgh, Pittsburgh, PA 15213, USA; ^4^Department of Speech and Language Pathology, Children's Hospital of Pittsburgh, Pittsburgh, PA 15213, USA

## Abstract

*Objective*. To determine best sedation protocol for videolaryngostroboscopy in children unable to tolerate non-sedated evaluation. *Materials and Methods.* Consecutive case series of 10 children with voice disturbances, unable to tolerate nonsedated videolaryngostroboscopy at an academic tertiary care children's hospital. Flexible fiberoptic videolaryngostroboscopy was performed and interpreted by pediatric otolaryngologist and speech and language pathologist. Sedation was administered with newly described protocol that allowed functional portion of evaluation. *Main Outcome Measures*: ability to follow commands and tolerate flexible fiberoptic videolaryngostroboscopy. *Secondary Outcome Measures*: total phonation time, complications, need for subsequent videolaryngostroboscopic attempts, clinical outcomes, and follow-up. *Results*. 10 children underwent procedure under conscious sedation. 9/10 children were able to perform simple tasks and maintain adequate phonation time to complete stroboscopic exam. 1/10 patients failed to complete exam because of crying during entire exam. Mean exam time was 2 minutes 52 seconds (SD 86 seconds), phonation time is 1 minute 44 seconds (SD 60 seconds), and number of tasks completed was 10.5 (SD 8.6). *Conclusions*. Conscious sedation for videolaryngostroboscopy can be safely and effectively performed in children unable to comply with nonsedated examination. Such studies provide valuable diagnostic information to make a diagnosis and to devise a treatment plan.

## 1. Introduction

Videolaryngostroboscopy is a valuable diagnostic modality currently used successfully in the adult population to offer diagnostic information regarding laryngeal disorders and voice abnormalities [1]. It is widely used in adults but its use in pediatrics is limited because of the inability of many children to tolerate the procedure [2, 3]. For children, the alternative to an office assessment is some type of conscious sedation protocol. The obvious limitation of conscious sedation is the loss of the child's capacity to follow commands and provide meaningful stroboscopic functional data to contribute to the diagnosis and management. A key question asked by pediatric otolaryngologists is whether or not the risk-benefit ratio of obtaining videostroboscopy in uncooperative children is favorable if conscious sedation is needed. A very recent publication provides an answer to this question. Mortensen et al. [4] published a retrospective series of 80 pediatric videostroboscopy patients, all of whom were previously examined by flexible laryngoscopy and treated with speech therapy for a presumed diagnosis of vocal cord nodules. The authors concluded that children with a history of prolonged dysphonia for whom treatment has failed should be referred for evaluation by videolaryngostroboscopy. Its value includes elucidation of subtle features of different disease processes: clarification of the differences between benign mucosal disorders that might require surgical intervention and assistance in identifying inflammatory processes that contribute to dysphonia. Finally, the only validated grading scale for pediatric vocal fold nodules is based on a transnasal videolaryngostroboscopic examination [5]. It is critical that a “universal language” be developed in order for clinicians to precisely communicate with one another and comparatively publish meaningful translational research. Clearly, for the standard of pediatric voice care to approximate that in adults, some alternative means of performing videolaryngostroboscopy is needed in children uncooperative for such an assessment in an office setting. 

A small number of series have demonstrated the effectiveness of rigid and flexible videolaryngostroboscopy in pediatric patients [2, 3]. Unlike the Mortensen study [4], what is unclear and not stated in these reports is the total number of children on whom videolaryngostroboscopy was attempted but failed in the voice clinic. Despite great effort in our voice center to familiarize and desensitize children to the equipment and procedure, some children are, nonetheless, unable to tolerate even a brief examination. It is likely that this limitation contributes to the fact that unlike in adults, pediatric videolaryngostroboscopy has failed to gain widespread acceptance. 

This study reports our conscious sedation protocol and results for successful videolaryngostroboscopy in children unable to tolerate the examination in the voice clinic while fully awake.

## 2. Materials and Methods

This study is a retrospective case series over 1 year. The protocol was reviewed by the hospital's Institutional Review Board (IRB) and approved by expedited review, in compliance with HIPAA guidelines (IRB no. PRO08050236). 

Consecutive children evaluated in the Voice, Resonance and Swallowing Center at the Children's Hospital of Pittsburgh of University of Pittsburgh Medical Center from June 2007 through June 2008 and undergoing videolaryngostroboscopy, as a diagnostic procedure, were included. All patients failed initial videolaryngostroboscopy as an outpatient procedure despite topical anesthesia and completion of a pediatric desensitization protocol. The overall rate of patients that underwent videolaryngostroboscopy in the operating room was 12% including patients that failed were noncompliant in the clinic as well as patients scheduled to undergo other procedures in the operating room for reasons unrelated to their voice problem. Informed consent was attained from parents or guardians in all instances.

All patients underwent the procedure with conscious sedation. Videolaryngostroboscopy was performed during spontaneous ventilation. A standardized sedation technique was employed. Midazolam (0.5 mg/kg po or 0.2 mg/kg intranasal) was given 20–30 minutes before transport to the endoscopy suite. After application of routine monitoring (pulse oximetry, EKG, and non-invasive blood pressure), a nitrous oxide (70%)/oxygen (30%) mixture was administered by mask to facilitate intravenous cannula insertion. Meperidine was then administered intravenously in increments (total dose: range 0.4–1.75 mg/kg). Because patient number 5 was adult sized, fentanyl (total dose 100 mcg) was substituted for meperidine. Simultaneously with the administration of meperidine, oxymetazoline was administered as a topical nasal decongestant and 1% lidocaine for topical anesthesia. Finally, propofol was administered, initially in bolus increments (total dose: range 0.42–2.63 mg/kg) for the first 8 patients; because of pain at the site of injection, the mode of administration was switched to a constant infusion (35–50 mcg/kg/min) for patients number 5, 9, and 10.

No jaw thrust, chin lift, continuous positive airway pressure, oral/nasal airways, or other airway maneuvers were used during the procedure. Spontaneous ventilation was preserved for the entire procedure. Lidocaine (1% for all but patient no. 5 who received 4%) was applied to the larynx. 

The main outcome measures were the child's ability to follow commands and tolerate flexible videolaryngostroboscopy. Secondary outcome measures included total phonation time, complications, a need for subsequent videolaryngostroboscopic attempts, clinical outcomes, and followup.

## 3. Results

10 children underwent videolaryngostroboscopy during the time period of the study. There were 6 females and 4 males. Mean age was 6 years (SD 3.7 years; range: 2 to 14 years)*. *The most common presentation was chronic or life long dysphonia. Flexible nasal endoscopy was attempted in clinic in 2 patients when the parents felt the child would cooperate for the exam and when the child was agreeable to the procedure. Both children however repeatedly pulled the endoscope out precluding completion of the evaluation. The remaining children were enrolled because their parents asserted in the voice clinic that they knew their children would not tolerate the procedure without sedation and, hence, were unwilling to consent.

Nine of ten children were able to complete the exam with adequate time of phonation for analysis and diagnosis. One of the 10 patients failed to complete the videolaryngostroboscopy; this was the same child that failed the flexible nasal endoscopy in clinic. The most common findings on subjective voice analysis preoperatively were harshness and breathiness associated with hyperfunction and strain. The most common diagnosis made with videolaryngostroboscopy was true vocal cord (TVC) nodules, found in 8/10 patients. The nodules were graded as described by Shah et al. in 2006 [5]. This 4-point grading scale based on videolaryngostroboscopic assessment for nodule size is as follows: Grade of 0 Normal/complete adduction with smooth vocal fold contour (i.e., no nodule present), Grade 1 Normal complete adduction with a small nodule located on the vibratory edge protruding less than 0.5 mm, Grade 2 nodules may be associated with an anterior glottic chink on adduction with a moderate sized nodule protruding > 0.5–1.0 mm, and Grade 3 nodules associated with an “hourglass” configuration of aperture closure on adduction with a large nodule protruding >1.0  mm on the vibratory edge. Additionally, the nodule was described as discrete by the subscript “D” when the base of the nodule was no more than twice the width or sessile, subscript “S”, where a broad base exceeded twice the width of the nodule. 

The mean exam time was 2 minutes 52 seconds (SD 86 seconds), phonation time, which represented total phonation time to diagnosis, is 1 minute 44 seconds (SD 60 seconds), and numbers of tasks completed were 10.5 (SD 8.6). Tasks include maximum phonation time, sustained vowel productions, mucosal wave assessment at low, medium, and high pitch and at different levels of intensity. Phase symmetry, amplitude, glottic aperture closure configuration, true vocal fold vertical height, and vocal cord morphology were readily assessed and documented.

The sedation technique was well tolerated in all children. Constant infusion of propofol was tolerated better because of decreased discomfort at the site of infusion. One child (patient 8) was initially over sedated but was able to be reversed to perform a full evaluation. Patient (6) who was crying for the entire duration of the exam still had evaluable mucosal waves which allowed assessment of nearly all stroboscopic metrics.

One of the children (not included in Table 1) was an autistic child with severe cognitive impairment who developed velopharyngeal insufficiency after adenoidectomy and did not respond to conservative medical management. The exam was limited to basic endoscopy to evaluate pharyngeal closure patterns with the child following similar commands with the same anesthetic protocol. This 14 y/o patient demonstrated a small (i.e., <2.0 cm) central gap/closure. Lateral wall movement was noted to be excellent as was palatal excursion. His total exam time was 1 minute and 59 seconds and phonation time was 1 minute and 41 seconds. By defining the pattern of closure and size of the central gap, surgical correction of his VPI was successfully planned and executed which would have otherwise been impossible without such a functional evaluation.

Our secondary outcome measures include complications, need for subsequent videolaryngostroboscopic attempts, clinical outcomes, and followup. There were no complications from the procedure and none needed additional attempts because all had complete evaluations in the operating room. Overall, all patients were treated according to their stroboscopic diagnoses.

## 4. Discussion

Pediatric voice disorders are not uncommon—with a range of incidence from 1% to 20% based on different series [6–8]. It is likely that as diagnostic and therapeutic methods improve and as awareness amongst primary care physicians increases, the incidence will increase. Evaluation of children with voice disorders is a challenge considering that examination of the larynx requires tolerance of an endoscopic visualization of the larynx [9]. Videolaryngostroboscopy is standard of care in adult laryngology and is critical to proper diagnosis and management. It offers evaluation of the larynx and any inherent structural changes, in addition to diagnosing gross pathology. It allows for an assessment of functional features not possible with conventional endoscopic techniques [2]. 

A study by Wolf et al. has shown that rigid stroboscopy can be safely and effectively implemented in children [3]. They found that the main factors for failure of the procedure were gag reflex, short phonation time, and epiglottic position in certain instances. The youngest child in this study was 6 years of age and the most common diagnosis was vocal cord nodules, followed by vocal cord cysts. They recommended limiting rigid stroboscopy in children over age of 10 years.

A more recent series by Hartnick and Zeitels describes their experience with flexible videolaryngostroboscopy in 25 children ranging from 19 months to 13 years with the mean age of 7 years [2]. They report successful exams in all 25 children using a 3.2 to 3.9 mm nasal flexible laryngostroboscopy. Their most frequent diagnosis was also vocal cord nodules. As noted in the introduction of this paper but worthy of emphasis is that the only validated grading scale for pediatric vocal fold nodules, the most common cause of dysphonia in children, is based on a transnasal videolaryngostroboscopic examination [5]. 

This study describes a successful algorithm for videolaryngostroboscopy in uncooperative children unable to tolerate the procedure in the Voice Center. Our algorithm sedates the child while preserving the child's capacity to fully cooperate with the phonatory tasks needed for such an assessment. In addition, the actual phonation and exam times were measured and documented, metrics heretofore unpublished in the literature. 

In the current series, there was only one child who did not tolerate the procedure yet a diagnosis was still made based on the laryngoscopic exam. Future research will focus on adjustments to the protocol to enable stroboscopy in such a patient as well as to report a larger sample size that reinforces this pilot proof-of-principal study. 

There are no current publications in the peer-reviewed literature specifically outlining a protocol for this highly specialized application, namely, pediatric videolaryngostroboscopy. In addition, this study introduces a novel algorithm for combining known medications, in a manner that is unique both in temporal administration as well as in the sequence of administration. Such an algorithm bridges the gap between the needs of the anesthesiologist and the otolaryngologist so that routine videolaryngostroboscopy can be added to the arsenal of diagnostic tools available to pediatric population.

## 5. Conclusions

We have successfully described and demonstrated a novel conscious sedation protocol that facilitates videolaryngostroboscopy in uncooperative children unable to tolerate the procedure in the voice center with excellent results. Future studies will test the algorithm using a prospective study design in a larger cohort of patients. Adjustments to the current protocol will also be explored for the extremely rare patient still unable to tolerate videolaryngostroboscopy.

## Figures and Tables

**Table 1:** Patient demographics and diagnoses.

Pt #	Age (yrs)	Presenting diagnosis	Diagnosis	Exam time (s)	Phonation time (s)
1	2	dysphonia	VC nodules	287	175
2	7	dysphonia	VC nodules	141	101
3	7	dysphonia	VC nodules	110	70
4	10	dysphonia	Plica ventricularis	271	177
5	5	dysphonia	VC nodules	0	0
6	3	dysphonia	VC nodules	103	53
7	3	dysphonia	VC nodules	127	92
8	4	dysphonia	VC nodules	225	187
9	5	dysphonia	VC nodules	143	87
